# Effects of Qigong Exercise on Fatigue, Anxiety, and Depressive Symptoms of Patients with Chronic Fatigue Syndrome-Like Illness: A Randomized Controlled Trial

**DOI:** 10.1155/2013/485341

**Published:** 2013-07-31

**Authors:** Jessie S. M. Chan, Rainbow T. H. Ho, Chong-wen Wang, Lai Ping Yuen, Jonathan S. T. Sham, Cecilia L. W. Chan

**Affiliations:** ^1^Centre on Behavioral Health, The University of Hong Kong, Hong Kong; ^2^Department of Social Work and Social Administration, The University of Hong Kong, Hong Kong; ^3^International Association for Health and Yangsheng, Hong Kong; ^4^Department of Clinical Onchology, Li Ka Shing Faculty of Medicine, The University of Hong Kong, Hong Kong

## Abstract

*Background*. Anxiety/depressive symptoms are common in patients with chronic fatigue syndrome- (CFS-) like illness. Qigong as a modality of complementary and alternative therapy has been increasingly applied by patients with chronic illnesses, but little is known about the effect of Qigong on anxiety/depressive symptoms of the patients with CFS-like illness. *Purpose*. To investigate the effects of Qigong on fatigue, anxiety, and depressive symptoms in patients with CFS-illness. *Methods*. One hundred and thirty-seven participants who met the diagnostic criteria for CFS-like illness were randomly assigned to either an intervention group or a waitlist control group. Participants in the intervention group received 10 sessions of Qigong training twice a week for 5 consecutive weeks, followed by home-based practice for 12 weeks. Fatigue, anxiety, and depressive symptoms were assessed at baseline and postintervention. *Results*. Total fatigue score [*F*(1,135) = 13.888, *P* < 0.001], physical fatigue score [*F*(1,135) = 20.852, *P* < 0.001] and depression score [*F*(1,135) = 9.918, *P* = 0.002] were significantly improved and mental fatigue score [*F*(1,135) = 3.902, *P* = 0.050] was marginally significantly improved in the Qigong group compared to controls. The anxiety score was not significantly improved in the Qigong group. *Conclusion*. Qigong may not only reduce the fatigue symptoms, but also has antidepressive effect for patients with CFS-like illness. Trial registration HKCTR-1200.

## 1. Introduction

CFS is characterized by unexplained persistent fatigue of at least 6 months with no definite effective treatment yet [1]. As a large part of the patients with CFS in the community remain unrecognized by general practitioners [2], CFS-like illness is defined based on self-reported fatigue symptoms and medical history with similar criteria for CFS, but no confirmed clinical examination [3–5]. Current and lifetime psychiatric disorders were common among the patients with CFS-like illness [6–9], with particularly strong association between unexplained fatigue and depression [10, 11]. A study with a multinational primary care sample from 14 countries suggested that over 80% of patients with CFS-like illness had a lifetime psychiatric disorder such as depression or generalized anxiety disorder [7, 12]. Most of the patients with CFS-like illness are undertreated for psychiatric illness [6]. Unexplained chronic fatigue is also a common disabling condition in the general population and is strongly associated with psychiatric morbidity [13]. In Hong Kong, the lifetime prevalence of anxiety and depressive disorders was 54% among the primary care patients with chronic fatigue (CF) [14]. The patients with CFS-like illness reported poorer mental health (higher levels of anxiety and depression) than their non-CFS-like illness counterparts [15].

To date, no curative treatment that is effective exists for the patients with CFS-like illness [16]. The use of complementary and alternative medicine (CAM) is increasing among the patients with CFS-like illness. A recent systematic review of 26 randomized clinical trials (RCTs) has suggested beneficial effects of CAM including Qigong, massage, and tuina for patients with CFS [17]. Qigong is an ancient self-healing mind-body exercise, which includes meditation, breathing, body posture, and gentle movement. It focuses to promote the circulation of vital energy, which is called “Qi” in the meridian system (Qi vital energy channel) of the human body to facilitate the harmony of the mind, body, and breathing [18].

 A number of empirical studies reported that Qigong had beneficial effects on fatigue symptoms [19, 20] and other outcomes related with CFS such as sleep, pain, mental attitude, and general mobility [21]. Our prior study demonstrated that Qigong exercise was effective in reducing the severity of fatigue symptoms, improving health-related quality of life [22], and increasing telomerase activity for the patients with CFS-like illness [23]. RCTs of Qigong exercise also suggested a beneficial effect of Qigong for older people with depressive symptoms secondary to chronic illnesses [24, 25]. However, a recent systematic review and meta-analysis of the effect of Qigong exercise on depressive and anxiety symptoms suggested that scientific evidence in the field was still limited, and that further rigorously designed RCTs were warranted [26]. To date, to our knowledge, no study has examined the effect of Qigong exercise on depressive and anxiety symptoms in patients with CFS-like illness. Thus, the purpose of this large-scale study was to investigate the effectiveness of Qigong exercise as a modality of complementary and alternative therapy in reducing fatigue, anxiety, and depressive symptoms of patients with CFS-like illness.

## 2. Methods

### 2.1. Study Participants

One thousand four hundred and forty-one Chinese adults who claimed to have fatigue symptoms volunteered to fill in an online questionnaire after the study was advertised in the media. The screening questionnaire was set according to the US Centers for Disease Control and Prevention (CDC) Diagnosis criteria for CFS [1], which is widely used in the field. As it was rare that patients with persistent fatigue symptoms alone stayed in public hospitals, the participants were recruited from local community.

 The diagnosis of CFS-like illness [3–5] was made based on subjective chronic symptoms and their medical history self-reported in the online questionnaire without further clinical confirmation by medical examination. A participant was diagnosed as having CFS-like illness if he or she had unexplained, persistent fatigue over 6 months which was of new onset (not lifelong) with presence of four or more of the following eight symptoms: impaired memory or concentration, postexertion malaise, unrefreshing sleep, muscle pain, multijoint pain, new headaches, sore throat, and tender lymph nodes [1]. To minimize the impact of other chronic illness as much as possible, those with any medical conditions that may explain the presence of chronic fatigue were excluded.

**Figure 1:**
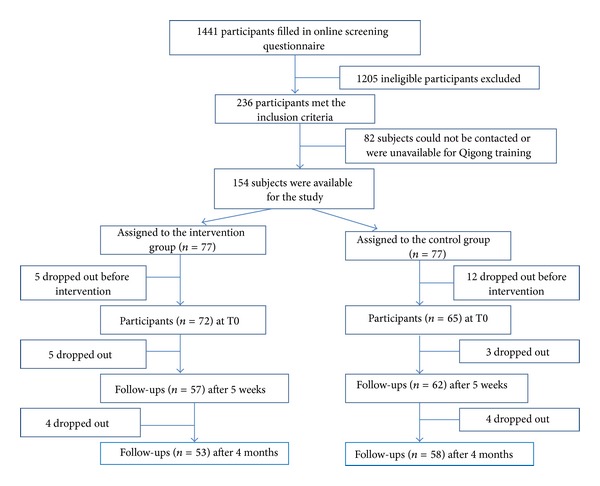
Flow chart of the selection of participants in the study.

 Two hundred and thirty-six participants met the inclusion criteria, of which 82 participants were excluded because they could not be contacted or were unavailable for the Qigong training. One hundred and fifty-four participants with CFS-like illness were recruited into the study and were randomly assigned to the intervention group (*n* = 77) and control group (*n* = 77), respectively. Among these 154 participants, 5 subjects in the intervention group and 12 subjects in the control group dropped out before the Qigong class. Only 137 subjects (72 for intervention group and 65 for control group) were included as the final sample for the data analysis. A flow chart of the selection of participants is presented in Figure 1.

### 2.2. Study Design and Procedure

This was a prospective randomized wait list-controlled trial. Each potential participant was required to complete an online screening questionnaire and was evaluated for eligibility by a pair of investigators with any discrepancies being resolved by discussion. Eligible participants were required to complete an additional questionnaire to measure the severity of their chronic fatigue symptoms and depressive and anxiety symptoms before intervention (T0) after having signed the written informed consent form. They were then randomly assigned to either an intervention group or a waitlist control group. Randomization was done using computer-generated random numbers. Blinding the participants to the allocation was not possible due to the nature of intervention. The intervention program lasted 4 months, with group Qigong training for 5 weeks followed by home-based Qigong exercise for 12 weeks in the intervention group. The primary outcome was fatigue symptoms and the secondary outcomes were anxiety and depressive symptoms. Data for the outcome measures were also collected at postintervention (T1) from each subject in the intervention group and control group. Ethical approval was obtained from the local review board.

Sample size was calculated according to power and estimated effect size. In order to achieve statistical power of 80% at a significance level of 0.05 (assuming treatment  effect = 3 and standard  deviation = 5 according to a previous local study on CFS [27]), 53 participants were required in each group. Assuming 30% dropout rate, at least 76 subjects were required in each group (the intervention group and the wait-list control group).

### 2.3. Intervention

Participants in the intervention group attended 10 sessions of Qigong exercise training (Wu Xing Ping Heng Gong, *五行平衡功*) twice a week for 5 consecutive weeks, followed by home-based Qigong self-practice for 12 weeks. Each session of Qigong exercise training lasted 2 hours, with a brief introduction of the basic theories of traditional Chinese medicine (such as the concepts of Qi, yin-yang, five elements, and meridian system) or the precautions in doing Qigong exercise including answering any questions or concerns raised by the participants about Qigong practice (45 min), followed by mindful meditation for relaxation and then gentle movement or body stretching in standing postures to facilitate a harmonious flow of Qi along the energy channels (15 min) and a 1 h session of Qigong exercise training, which was delivered by an experienced Taoist Qigong master (Yuen L. P.) with more than 20 years of experience in Qigong practice and also a background in traditional Chinese Medicine.

Apart from mindful meditation, rhythmic breathing and concentrated relaxation, Xu Xing Ping Heng Gong, was applied in this study including 10 forms of movement which aims at enhancing the smooth flow of Qi along the various meridians of the body and meditation for relaxation and mind concentration. The movements involve stretching of arms and legs, turning of torso, relaxing, and deep breathing with the objectives of fostering harmonious energy flow of Qi along the various meridians of the body. A description of the Xu Xing Ping Heng Gong is presented in Appendix. 

All participants in the intervention group were also required to do Qigong self-practice for at least 30 minutes every day at home during the 4-month intervention period. To assess home exercise, they were required to report the frequency and duration as well as adverse effects of the self-practice at home at the end of the program. The participants in control group were advised to keep their lifestyle as usual and to refrain from joining any outside Qigong exercise class during the study period. No participants in the control group joined any outside Qigong class as they were provided the Qigong training after the final outcome measurements were collected.

### 2.4. Measurements

#### 2.4.1. Screening Measures

The potential participants were screened by online questionnaire including (1) whether or not the fatigue symptoms persisted or relapsed for six or more months; (2) a list of eight chronic fatigue symptoms of CDC diagnostic inclusion criteria for CFS [1]; (3) a list of medical diseases based on the CDC diagnostic exclusion criteria for CFS [1] according to their self-reported medical history without further medical examination; (4) basic demographic data such as age, gender, employment status, education level, marital status, religion, and monthly income; (5) lifestyle including exercise habits, smoking, alcohol drinking, and sleep time.

#### 2.4.2. Chalder Fatigue Scale

The severity of fatigue symptoms was measured by the Chalder Fatigue Scale, which is a 14-item self-rating scale to measure the severity of both physical fatigue symptoms (8 items) and mental fatigue symptoms (6 items). The response pattern for each item is a five-point Likert scale (none, better than usual, no more than usual, worse than usual, much worse than usual), which is scored from 0 to 4. The subscale scores are equal to the summed scores of all items in the subscale and the total fatigue score was obtained by adding up all of the 14 items (the higher, the worse) [28]. The Chinese version of the Chalder Fatigue Scale has shown acceptable psychometric properties [29].

#### 2.4.3. Hospital Anxiety and Depression Scale (HADS)

Depressive and anxiety symptoms were measured by the HADS [30], which is a 14-item instrument with two subscales measuring anxiety symptoms (7 items) and depressive symptoms (7 items) separately. Each item is scored on a 0–3 scale and the total score of each subscale is scored on a 0–21 scale, with a higher score indicating a higher level of anxiety and depressive symptoms. Internal consistency for HADS Chinese version was revealed to be satisfactory, with Cronbach's alpha coefficients of 0.77 for anxiety subscale and 0.82 for depression subscale, respectively [31, 32].

### 2.5. Statistical Analyses

Means and standard deviations were used to summarize continuous data and frequency was used to summarize categorical data. Differences at baseline for the demographic information, lifestyles, and reported fatigue, anxiety, and depressive symptoms between the two groups were compared using a *t*-test for continuous data and a Chi-squared test for categorical data. The within group effects of outcome measures were compared between pre- and postintervention using pairwise *t*-test for each group. The effect size was determined by Cohen's d statistics for each outcome. The repeated measures analyses of variance (ANOVA) were then conducted to assess the interaction effect of group and time for each outcome. Intention to treat analysis was applied in this study and the missing data were substituted by the last observed values. The correlation analysis of the changes in all outcomes between pre- and postintervention and the linear regression analysis using the change of depression score as a dependent variable and changes of other outcomes as independent variables were also conducted. All data analysis was conducted with Statistical Package for the Social Sciences (SPSS version 18.0, SPSS Inc., Chicago, IL, USA). A *P* value of less than 0.05 was considered as statistically significant.

## 3. Results

### 3.1. The Demographic Characteristics and Lifestyles at Baseline

The data on demographic characteristics and lifestyles of the two groups are shown in Table 1. The mean ages were 42.4 (SD = 6.7) in the intervention group and 42.5 (SD = 6.4) in the control group, respectively. More than 70% of the participants were female (72% and 82% in the intervention and control groups, resp.). As shown in the table, baseline characteristics were well balanced between the two groups. The average number of reported fatigue symptoms was 6.3 (SD = 1.4) in both groups. Among eight chronic fatigue symptoms (last at least 6 months), the most common symptoms (*n* = 129, 94.2%) was sleep disturbance followed by muscle pain (*n* = 128, 93.4%) and impaired memory/concentration (*n* = 126, 92.0%). There was no significant difference in fatigue symptoms between the two groups. Overall, the participants had a moderate level of anxiety symptoms (mean scores for the anxiety subscale were 11.0 for the intervention group and 10.9 for the control group resp.) and a mild level of depressive symptoms (mean scores for the depression subscale were 9.1 and 9.4 for the intervention and control groups resp.) at baseline.

### 3.2. The Efficacy of Intervention

Table 2 shows the within-group and between-group differences of fatigue symptoms as measured by the Chalder Fatigue Scale and anxiety and depressive symptoms as measured by the HADS for the two groups. At baseline (T0), two groups were comparable in terms of total fatigue score, physical fatigue score, mental fatigue score, anxiety score, and depression score (*P* > 0.05 for all variables). Compared with baseline values, the total fatigue score (*d* = − 1.2, *P* < 0.001), physical fatigue score (*d* = − 1.4, *P* < 0.001), mental fatigue score (*d* = − 0.9, *P* < 0.001), anxiety score (*d* = − 1.1, *P* < 0.001), and depression score (*d* = − 0.5, *P* < 0.001) were significantly improved in the intervention group after 4 months of Qigong intervention, while the total fatigue score, physical fatigue score, mental fatigue score and anxiety score in the control group were also significantly improved 4 months after (*d* = − 0.8, *P* < 0.001; *d* = − 0.8, *P* < 0.001; *d* = − 0.6, *P* < 0.001; *d* = − 0.6, *P* = 0.006, resp.). However, the change of the depression score in the control group was not significant (*d* = 0.1, *P* = 0.365).

The between-group difference in the change of each outcome measure was then examined by interaction effect of time and group. Compared with controls, the total fatigue score [*F*(1,135) = 13.888, *P* < 0.001], physical fatigue score [*F*(1,135) = 20.852, *P* < 0.001], and depression score [*F*(1,135) = 9.918, *P* = 0.002] were significantly improved, and the mental fatigue score [*F*(1,135) = 3.902, *P* = 0.050] was marginally significantly improved in the intervention group, whereas the change in the anxiety score in the intervention group was not significant after adjusting for control [*F*(1,135) = 0.302, *P* = 0.584]. No adverse effects were reported in both groups during the implementation of intervention and self-practice at home throughout the study.

### 3.3. Predictors of Changes in Depressive Symptoms

In correlation analysis, change in the depression score was significantly correlated with changes in the total fatigue score (*r* = 0.331, *P* < 0.001) and anxiety score (*r* = 0.579, *P* < 0.001). Linear regression analysis further revealed that the change in the total fatigue score (*β* = 0.182, *P* = 0.013) and anxiety score (*β* = 0.528, *P* < 0.001) significantly explained the change in the level of depressive symptoms (adjusted *R* ^2^ = 0.356).

## 4. Discussion

To the best of our knowledge, this study is the first large-scale randomized control trial to investigate the anti-depressive effect of Qigong exercise for the patients with CFS-like illness. The findings of this study showed that Qigong exercise could improve depressive symptoms and fatigue symptoms among the patients with CFS-like illness, which provided additional evidence to support the conclusive statement of a recent systematic review [26] that Qigong exercise may have beneficial effect on depressive symptoms. An earlier study [33] showed that depressive symptoms were not significantly improved after Qigong intervention in elderly with chronic illnesses, probably due to the small sample size (*n* = 50) and short intervention period (12 weeks). The current study with a larger sample suggested that Qigong exercise could reduce depressive symptoms for persons with CFS-like illness. Our findings coincided with the results reported in other studies that Qigong exercise might have a beneficial effect on depressive symptoms in depressed elderly with chronic illness [24, 25], mild essential hypertension [34], subhealth [35], and female college students [36].

In this study, participants' anxiety symptoms were significantly improved in both groups compared with baseline values, but there was no significant difference in the change of anxiety symptoms between the intervention group and the control group. To date, only a very few studies [34–37] have examined the effect of Qigong exercise on anxiety symptoms but the findings were inconsistent, probably due to diversity of participants or sample size, variability in the severity of comorbidities or anxiety symptoms, and heterogeneity in outcome measures. Our results supported the conclusive statement of a recent systematic review that the limited existing evidence did not support the effect of Qigong exercise on anxiety symptoms [26]. Further well-designed RCTs were still warranted to test the effect of Qigong on anxiety disorders. 

Interestingly, we found that the total fatigue, physical fatigue, mental fatigue, and anxiety symptoms in the waitlist control group were also significantly improved four months after. These results may be explained by two schools of mechanism. The first one may be that the results were due to the effects of self-care or other self-applied treatments. Generally, efforts to manage their symptoms are always under way for patients with chronic illnesses. In our study, most participants reported that they had tried other numerous therapies to manage their symptoms or treat their illnesses before joining this study, even though those therapies were ineffective. The second possible reason may be related to a beneficial effect of hope on physical health and psychological or emotional wellbeing [38]. In our study, all participants in the control group were told that they could join the Qigong training after completing the study, so they might have a desirable expectation that might exert a beneficial effect on their psychological wellbeing and physical symptoms. Previous studies have shown that hope is inversely associated with total fatigue, mental fatigue and level of anxiety and depression [39–41].

Our study also showed a significant correlation between alleviation of depression and fatigue reduction, as well as reduced anxiety following Qigong exercise. Regression analyses further revealed that the improvements of fatigue and anxiety symptoms significantly predicted the alleviation of depressive symptoms after Qigong intervention. The results confirmed an established association between fatigue symptoms and psychiatric disorders [8, 9, 11].

Qigong as a mind-body integrative exercise is distinguished from conventional forms of exercise [42]. The underlying physiological mechanism of mind-body intervention may be of interest. Tsang and Fung [43] have hypothesized three possible neurobiological pathways of the anti-depressive effect of Qigong exercise including monoamine neurotransmitters in the brain, the hypothalamic-pituitary-adrenal (HPA) axis, and the brain-derived neurotropic factors (BDNF), but these hypotheses need to be further tested. 

Although the results of our study are promising, some limitations of this study should be noted. First, the participants with CFS-like illness were recruited from local community, who did not receive medical examinations conducted by clinicians. Thus, some of them may not fully meet the CDC criteria for CFS. Although around three-quarters of the participants were female, it is similar to the proportion of females with CFS in other earlier studies [16]. Second, this study was a waitlist controlled trial, so social interaction effects might have been existed in the intervention group. It is recommended that active controls should be applied in future studies to avoid possible placebo effect. Third, the dosage and quality of home-based Qigong exercise were not adjusted for in our data analysis. Given that some studies have suggested a relationship between amount of Qigong practice and health outcomes [44], it should be measured and taken into account in data analysis in future studies. Finally, some other factors such as diet, physical activities, social interaction, body weight, and comorbidities may affect the outcomes, which should be adjusted in further trials. Despite these limitations, this study was the first RCT to examine the effect of Qigong exercise on anxiety and depressive symptoms among patients with CFS-like illness, which may provide complementary evidence to the body of knowledge in this field.

## 5. Conclusion

In conclusion, the results of this study show that Qigong exercise may be effective in reducing fatigue symptoms and alleviating depressive symptoms for patients with CFS-like illness and that the improvement of fatigue symptoms may predict the alleviation of depressive symptoms after Qigong intervention. The findings suggest that Qigong exercise may be used as an alternative and complementary therapy or rehabilitation program for patients with CFS-like illness.

## Figures and Tables

**Table 1:** Patients' demographic information and lifestyles at baseline (*n* = 137).

Demographic	Intervention (*n* = 72)		Control (*n* = 65)		P*
	Mean (SD)	*N* (%)	Mean (SD)	*N* (%)	
Age (years)	42.4 (6.7)		42.5 (6.4)		.979
Gender					.198
Female		52 (72.2%)		53 (81.5%)	
Employment					.629
Full-time		55 (76.4%)		52 (80.0%)	
Part-time		3 (4.2%)		1 (1.5%)	
Housewife		9 (12.5%)		10 (15.4%)	
Unemployed		4 (5.6%)		1 (1.5%)	
Other		1 (1.4%)		1 (1.5%)	
Education					.366
Secondary school		31 (43.1%)		33 (50.8%)	
Tertiary or above		41 (56.9%)		32 (49.2%)	
Marital status					.738
Single		21 (29.2%)		23 (35.4%)	
Married/cohabiting		46 (63.9%)		38 (58.5%)	
Divorced/separated/widowed		5 (6.9%)		4 (6.2%)	
Have religion					.334
Yes		21 (29.2%)		24 (36.9%)	
Monthly income					.824
<10,000		11 (15.3%)		6 (9.2%)	
10,000–19,999		20 (27.8%)		18 (27.7%)	
20,000–29,999		9 (12.5%)		8 (12.3%)	
≥30,000		9 (12.5%)		10 (15.4%)	
No income/not available		10 (13.9%)		7 (10.8%)	
Not want to answer		13 (18.1%)		16 (24.6%)	
Lifestyles					
Do exercise regularly		19 (26.4%)		17 (26.2%)	.975
Smoking		6 (8.3%)		2 (3.1%)	.190
Alcohol drinking		31 (43.1%)		22 (33.8%)	.269
Sleep time (hours)	5.0 (1.8)		4.7 (2.2)		.434
Average number of reported fatigue symptoms	6.3 (1.4)		6.3 (1.4)		.864

*Chi-squared test for categorical variable and *t*-test for continuous variable.

**Table 2:** Within-group and between-group comparisons for Chalder Fatigue Scale, anxiety, and depression at T0 and T1 (*n* = 137) using repeated measures ANOVA.

	Within-group effects				Between-group effects		
	Baseline (T0)^ a^	Post-intervention (T1)^b^			T1-T0	Time × group	
	Mean (SD)	Mean (SD)	*P* ^b^	Effect Size (*d*)	Mean (SD)	*F*(1,135)	*P *
Total fatigue score						13.888	.000
Intervention group (*n* = 72)	39.7 (6.6)	26.6 (13.6)	<0.001	−1.2	−13.1 (11.7)		
Control group (*n* = 65)	39.8 (6.3)	33.2 (9.6)	<0.001	−0.8	−6.6 (8.3)		

Physical fatigue score						20.852	.000
Intervention group (*n* = 72)	24.7 (4.0)	15.9 (8.0)	<0.001	−1.4	−8.8 (7.3)		
Control group (*n* = 65)	24.6 (3.7)	20.8 (5.7)	<0.001	−0.8	−3.8 (5.0)		

Mental fatigue score						3.902	.050
Intervention group (*n* = 72)	15.0 (3.8)	10.6 (6.1)	<0.001	−0.9	−4.3 (5.3)		
Control group (*n* = 65)	15.2 (3.9)	12.4 (4.9)	<0.001	−0.6	−2.7 (3.9)		

Anxiety score						0.302	.584
Intervention group (*n* = 72)	11.0 (2.1)	8.7 (3.2)	<0.001	−1.1	−2.3 (3.9)		
Control group (*n* = 65)	10.9 (2.4)	9.0 (4.0)	0.006	−0.6	−1.9 (5.4)		

Depression score						9.918	.002
Intervention group (*n* = 72)	9.1 (2.0)	7.7 (3.2)	<0.001	−0.5	−1.3 (2.7)		
Control group (*n* = 65)	9.4 (2.2)	9.8 (4.1)	0.365	0.1	0.4 (3.7)		

^a^Compared with control group using independent *t*-test, ^b^Compared with baseline using pairwise *t*-test.

## Appendix

### Description of the Movements in Wu Xing Ping Heng Gong

*Warm-up Movement. *Swinging of arms by turning the torso with relaxed shoulders (preferably to be practiced in a relaxing outdoor space with trees).

*Movement 1*. Standing on toes with hand movements to the front and to the side.

*Movement 2*. Circular movements of hands, wrists, hips; stretching by arching backwards of neck and torso.

*Movement 3*. Movement of fingers, wrists, elbow and shoulders; stretching of arms.

*Movement 4. *Movement of wrists; stretching shoulder muscles; twisting movements of shoulders.

*Movement 5. *Massage of ears.

*Movement 6. *Swinging of hands to gently hit the chest and back; standing on one foot and hitting back of the standing foot's calf by the dorsum of other foot.

*Movement 7. *Stretching of trunk and hip joints by stepping forward and backward.

*Movement 8. *Swinging movements of lower body; squatting and bending forward to stretch the back of the torso.

*Movement 9. *Movement of legs with hands in cupping pose; turning of torso in kneeling position.

*Sitting meditation. *Sitting meditation with deep breathing can be conducted for 20–30 minutes after the movement exercises if possible. If not, move directly to the concluding movement. Sitting meditation is recommended in the evening, before going to bed.

*Concluding movement. *Hands in cupping pose in front of the lower abdomen for about 20 seconds; rub hands and then use palms to massage face (upward movement like washing face), followed by the use of fingertips to massage the scalp in combing movement.
